# Targeting *Acanthamoeba* proteins interaction with flavonoids of Propolis extract by *in vitro* and *in silico* studies for promising therapeutic effects

**DOI:** 10.12688/f1000research.126227.1

**Published:** 2022-11-08

**Authors:** Imran Sama-ae, Suthinee Sangkanu, Abolghasem Siyadatpanah, Roghayeh Norouzi, Julalak Chuprom, Watcharapong Mitsuwan, Sirirat Surinkaew, Rachasak Boonhok, Alok K. Paul, Tooba Mahboob, Najme Sadat Abtahi, Tajudeen O. Jimoh, Sónia M.R. Oliveira, Madhu Gupta, Chea Sin, Maria de Lourdes Pereira, Polrat Wilairatana, Christophe Wiart, Mohammed Rahmatullah, Karma G. Dolma, Veeranoot Nissapatorn

**Affiliations:** 1 Department of Medical Technology, School of Allied Health Sciences and Center of Excellence Research for Melioidosis and Microorganisms (CERMM), Walailak University, Nakhon Si Thammarat, Thailand; 2 School of Allied Health Sciences, Southeast Asia Water Team (SEA Water Team) and World Union for Herbal Drug Discovery (WUHeDD), Walailak University, Nakhon Si Thammarat, Thailand; 3 Ferdows School of Paramedical and Health, Birjand University of Medical Sciences, Birjand, Iran; 4 Department of Pathobiology, Faculty of Veterinary Medicine, University of Tabriz, Tabriz, Iran; 5 Akkhraratchakumari Veterinary College and Research Center of Excellence in Innovation of Essential Oil, Walailak University, Nakhon Si Thammarat, Thailand; 6 Department of Medical Technology, School of Allied Health Sciences and Research Excellence Center for Innovation and Health Products (RECIHP), Walailak University, Nakhon Si Thammarat, Thailand; 7 School of Pharmacy and Pharmacology, University of Tasmania, TAS, Australia; 8 Department of Clinical Biochemistry, Faculty of Medicine International Campus, Shahid Sadoughi University of Medical Sciences, Yazd, Iran; 9 Department of Pharmacognosy and Pharmaceutical Botany, Faculty of Pharmaceutical Sciences, Chulalongkorn University, Bangkok, Thailand; 10 Department of Biochemistry, Faculty of Health Sciences, Islamic University in Uganda, Kampala, Uganda; 11 Hunter Medical Research Institute, NSW, Australia; 12 CICECO-Aveiro Institute of Materials, University of Aveiro, Aveiro, Portugal; 13 Department of Pharmaceutics, Delhi Pharmaceutical Sciences and Research University, New Delhi, India; 14 Faculty of Pharmacy, University of Puthisastra, Phnom Penh, Cambodia; 15 Department of Medical Sciences, University of Aveiro, Aveiro, Portugal; 16 Department of Clinical Tropical Medicine, Faculty of Tropical Medicine, Mahidol University, Bangkok, Thailand; 17 Institute for Tropical Biology & Conservation, University Malaysia Sabah, Sabah, Malaysia; 18 Department of Biotechnology & Genetic Engineering, University of Development Alternative, Dhaka, Bangladesh; 19 Department of Microbiology, Sikkim Manipal Institute of Medical Sciences, Sikkim Manipal University, Sikkim, India

**Keywords:** Propolis extract, anti-Acanthamoeba activity, encystation, pinocembrin, molecular docking, and dynamic simulation

## Abstract

**Background** **:** Propolis is a natural resinous mixture produced by bees. It provides beneficial effects on human health in the treatment/management of many diseases. The present study was performed to demonstrate the anti- *Acanthamoeba* activity of ethanolic extracts of Propolis samples from Iran. The interactions of the compounds and essential proteins of *Acanthamoeba* were also visualized through docking simulation.

**Methods: **The minimal inhibitory concentrations (MICs) of Propolis extract against *Acanthamoeba* trophozoites and cysts was determined *in vitro*. In addition, two-fold dilutions of each of the agents were tested for encystment, excystment and adhesion inhibitions. Three major compounds of Propolis extract such as chrysin, tectochrysin and pinocembrin have been selected in molecular docking approach to predict the compounds that might be responsible for encystment, excystment and adhesion inhibitions of *A. castellanii*. Furthermore, to confirm the docking results, molecular dynamics (MD) simulations were also carried out for the most promising two ligand-pocket complexes from docking studies.

**Results** **: **The minimal inhibitory concentrations (MICs) 62.5 and 125 µg/mL of the most active Propolis extract were assessed in trophozoites stage of *Acanthamoeba* *castellanii* ATCC30010 and ATCC50739, respectively. At concentrations lower than their MICs values (1/16 MIC), Propolis extract revealed inhibition of encystation. However, at 1/2 MIC, it showed a potential inhibition of excystation and anti-adhesion. The molecular docking and dynamic simulation revealed the potential capability of Pinocembrin to form hydrogen bonds with *A*. *castellanii* Sir2 family protein (AcSir2), an encystation protein of high relevance for this process in *Acanthamoeba*.

**Conclusions** **: **The results obtained provided a candidate for the development of therapeutic drugs against *Acanthamoeba* infection. *In vivo* experiments and clinical trials are necessary to support this claim.

## Introduction

*Acanthamoeba*, a free-living ameba, is a causative agent of fatal granulomatous amoebic encephalitis (GAE), *Acanthamoeba* keratitis (AK), *Acanthamoeba* pneumonia (AP), cutaneous acanthamoebiasis, and disseminated acanthamoebiasis found in humans ^1^. In healthy individuals with contact lenses, *Acanthamoeba* keratitis is increasingly being recognized as a serious sight-threatening ocular infection in public health worldwide ^2^. *Acanthamoeba* life cycle includes an active trophozoite stage and a dormant cyst stage. The trophozoite stage is the motile form that acquires nutrients, neutral pH, adequate food supply, ambient temperature, and balanced osmolality, while the cyst is triggered by extreme conditions, such as food crisis, hyper- or hypo-osmolarity, temperature, and excessive acid to basic conditions. Regarding the *Acanthamoeba* keratitis, the cyst form can be found in the acceptor cornea and is difficult to treat due to the resilient nature of the cyst. Current treatment regimens usually include standard anti- *Acanthamoeba* drugs, biguanide and diamidine, for an effective treatment against cysts ^3^. However, long-term treatment has also been suggested to induce a resistant *Acanthamoeba* cyst form due to a non-specific symptom at the early stage of AK, which share other common features, such as eye pain and redness ^4^.

Propolis or bee glue is a mixture of honeybees and natural products of different parts of plants ^5^ that is used for the construction and repairing beehives. Propolis hardens the cell wall of beehives, contributes to an aseptic internal environment ^6^, and acts as a protective barrier against predators. In addition, Propolis property contains several biological activities such as anti-inflammation, anti-proliferation, antioxidant, anti-diabetic activity, and antimicrobial activities ^7– 9^.

Therefore, this study sought to evaluate an amoebicidal activity and anti- *Acanthamoeba* encystation, excystation and anti-adhesion by Propolis extract that could offer an alternative treatment strategy for *Acanthamoeba* infection. Molecular docking simulation was included to predict a predominant binding mode of small molecules derived from Propolis with essential proteins from *Acanthamoeba* spp., to identify a relevant stable protein-ligand complex for future drug development.

## Methods

### Preparation extracts

Three Propolis samples were collected from Sardasht county, Boroujen city and Kermanshah city from Iran. The raw materials of Propolis were cut into small pieces, homogenizing (20 g) with 50 mL absolute ethanol and incubated at room temperature for seven days without shaking. Then, the extract was filtered through Whatman No. 1 filter paper, and the alcoholic extract was evaporated under vacuum with a rotary evaporator until it was dry. Dried extracts were preserved at 4°C and re-suspended in dimethyl sulfoxide (DMSO) at 100 mg/mL concentration before use.

### Culture of *Acanthamoeba castellanii*

*Acanthamoeba castellanii* non-pathogenic strain (ATCC 30010) and *Acanthamoeba castellanii* pathogenic strain (ATCC 50739) were obtained from the American Type Culture Collection (ATCC). Trophozoites were grown in 75 cm ^2^ tissue culture flasks in Peptone Yeast Extract Glucose Broth (PYG) medium containing proteose peptone 0.75% (w/v), yeast extract 0.75% (w/v) and glucose 1.5% (w/v) (purchased from HiMedia Laboratories Pvt.Ltd., Mumbai, India), without shaking at 28°C as described previously ^10^. For cysts, trophozoites were transferred from the PYG medium to the Neff’s encystment medium (NEM) containing 0.1 M KCl, 8 mM MgSO _4_·7H _2_O, 0.4 mM CaCl _2_·2H _2_O, 1 mM NaHCO _3_, 20 mM ammediol (purchased from RCI Labscan Limited, Bangkok, Thailand) and were cultured in this medium for seven days to obtain mature cysts. After that, mature cysts were harvested and washed twice using 10 mL sterile phosphate-buffer saline (PBS).

### Determination of minimal inhibitory concentration (MIC)

The MIC was determined by the micro-dilution method using serially diluted (two-fold) Propolis extracts. Determination of the MIC of the Propolis extract was examined according to a previous study ^10^. Stock solution of extracts (4 μL) were transferred into the first well of 96-well microplates, including 196 μL PYG medium to obtain a final concentration of 2,000 µg/mL. A two-fold serial dilution of the extracts were prepared in 96-well assay microplates to obtain concentrations in the range of 7.8–1,000 μg/mL in PYG medium. Then, 100 µL trophozoites or cysts (2×10 ^5^ cells/mL) were added. The final volume in each well was 200 μL. Plates were incubated for 24 hours at 28°C. The percentage of cell viability was determined using 0.2% trypan blue, obtained by manual counting under inverted microscopy (Nikon, Tokyo, Japan). The relative percentage of parasite viability was defined as: (mean of the treated parasite/mean of the control) × 100. The lowest concentration of extract that inhibited 90% of *A. castellanii* growth was recorded as the MIC. The commercial antibiotic agent, chlorhexidine was used as positive control, while 1% DMSO was used as negative control.

### Anti-encystation on *Acanthamoeba castellanii*

Anti-encystation was performed as previously studied ^11^ with modifications. Briefly, *Acanthamoeba* trophozoites (5×10 ^5^ cells/mL) were incubated in Neff’s medium in a 96-well plate containing Propolis extracts at different concentrations (1/2 MIC, 1/4 MIC, 1/8 MIC, 1/16 MIC). Plates were incubated at 28°C for seven days, and the total amoebae number was counted using a hemocytometer (Boeco, Hamburg, Germany). Following the sodium dodecyl sulfate (SDS, 0.5% final concentration) was added and incubated for 1 hour to dissolve trophozoites and immature cyst. The remaining cysts were counted using a hemocytometer after the addition of SDS. To quantify encystation, the percentage of *Acanthamoeba* encystation was determined as follows: (total number of amoebae post-SDS treatment/total number of amoebae pre-SDS treatment) × 100. Phenylmethylsulfonyl fluoride (PMSF) (10 mM final concentration) was used as a positive control, whereas 1% DMSO was used as a negative control.

### Anti-excystation on *Acanthamoeba castellanii*

For excystation, *Acanthamoeba* cysts (5×10 ^5^ cells/mL) were incubated with various concentrations of Propolis extracts (1/2 MIC, 1/4 MIC, 1/8 MIC, 1/16 MIC) in PYG medium in 96-well plate at 28°C for seven days ^12^. The effects of the extract on excystation were observed under an inverted microscope. The total amoebae were counted using a hemocytometer while SDS (0.5% final concentration) was added and incubated for 1 hour to dissolve trophozoites and immature cysts. The remaining cysts were counted after the addition of SDS. To quantify excystation, the percentage of *Acanthamoeba* excystation was determined as follows: (total number of amoebae pre-treatment with SDS − total number of amoebae post-SDS treatment)/total number of amoebae pre-SDS treatment) × 100. PMSF (10 mM final concentration) and amoebae alone were used as control.

### Anti-adhesion on *Acanthamoeba castellanii*

The anti-adhesion assay was modified as previously reported ^13^. Trophozoites (4 × 10 ^5^ cells/mL) were added to each well of a 96-well polystyrene microtiter plate supplemented with 1/2 MIC, 1/4 MIC, 1/8 MIC, 1/16 MIC of Propolis extract. Plates were incubated at 28°C without shaking for 24 hours. After incubation, a removing step to discard unbound trophozoites was performed. Plates were washed once with 0.1 M PBS, then air dried for 30 minutes at room temperature. The wells were stained with 0.1% crystal violet assay for 30 minutes. The crystal violet was eliminated, and the plates were washed with water and air dried. An aliquot of DMSO was added to the well and the absorbance was read at OD _570_ nm. Wells containing trophozoites without compounds or chlorhexidine were used as controls. The percentage of inhibition was calculated by following the formula: percentage of inhibition = (control OD – test OD/control OD) × 100.

### Cytotoxicity assay

The cytotoxic effects of the most active Propolis extract were evaluated using the Vero cell line (ECACC 84113001, RRID:CVCL_0059). Cells were cultured in Dulbecco’s Modified Eagle’s medium (DMEM) (Merck KGaA, Darmstadt, Germany) supplemented with 10% FBS (Sigma Aldrich, St. Louis, USA), and 1% antibiotic containing penicillin G (100 units/mL) and streptomycin (100 μg/mL). The culture was incubated at 37°C, humidified with 5% CO _2_ in an incubator (non-shaking). After the cells reached 90% confluence, the detachment was performed with trypsin and ethylene diamine tetra-acetic acid (EDTA) and incubated at 37°C in 5% CO _2_. Single cells at a density of 1.5 × 10 ^4^ cells/100 μL were seeded into each well of a 96-well polystyrene plate and allowed to attach for 24 hours. Then, 100 μL propolis extract, eye drops, and combined set were gently added. After incubation for 24 hours, the cytotoxic effects were determined using an MTT assay ^14, 15^. The absorbance was measured using a microplate reader (Biotek, Cork, Ireland) at 570 nm. The survival percentage was calculated using the following equation:


%survival=(ABt/ABu)×100

ABt and ABu denote the absorbance values of treated and untreated cells, respectively.

### Gas chromatography-mass spectrometry (GC-MS) analysis

Propolis extract (20 mg/mL) was diluted in ethanol (1:10), the solution was centrifuged for 10 min at a speed of 10,000 rpm at temperature of 10°C. The solution was used for analysis. GC-MS analysis was performed using Agilent Technology 7890 A (GC) equipped with 5977A Mass Selective Detector (MS) (Agilent, California, USA). A VF-WAXms capillary column of dimensions 30 m × 250 × 0.25 μM was used with helium gas as the carrier at 30 m × 250 × 0.25 μM at a flow rate of 1 mL/min. The column temperature was initially programmed at 60°C, which was increased to 160°C at 10°C/minute and further increased to 325°C at 2.5°C/minute, hold time for 15 minutes. The mass spectra was collected at 70 eV ionization voltage over the range of *m/z* 35 to 500 in full scan mode. Chemical constituents were identified by comparing their mass spectral data with those from the Wiley library.

### Data analysis

The experiments were repeated in triplicate. All data were recorded and entered into IBM SPSS Statistics (RRID:SCR_016479) version 26.0 (SPSS Inc. Chicago, IL, USA). Data were expressed as mean ± SD. Statistical analysis was conducted using a two-tailed unpaired Student’s t-test. p < 0.05 was considered statistically significant in all analyses.

### The three-dimensional (3D) structures prediction

The effect of Propolis compounds on essential proteins of *A. castellanii* was investigated using the computational modelling method. This study focuses on three critical proteins: the Sir2 family protein, the mannose-binding protein, and the G protein-coupled receptor. The I-TASSER server was used to predict the 3D structures of these proteins ^16, 17^. FASTA sequences of *A. castellanii* Sir2 family protein (AcSir2) (NCBI Reference Sequence: XP 004358245.1) ^18^, *A. castellanii* mannose-binding protein (AcMBP) (GenBank: AAT37865.1) ^19^, and *A. castellanii* G protein-coupled receptor (AcGPCR) (GenBank: ELR16814.1) ^18^ were used as inputs, with no constraints or applied templates. The most confidently predicted model was constructed using the most significant templates in the threading alignments. Then, the quality of the predicted 3D model was further improved using ModRefiner ^20^. Finally, the stereochemical quality of the protein structures was determined using PROCHECK (RRID:SCR_019043) ^21^.

### Preparation of protein and ligand structures for molecular docking

In this study, we used molecular docking to measure the binding energies of major compounds of Propolis such as pinocembrin, chrysin, and tectochrysin to those of *A. castellanii* essential proteins such as AcSir2, AcMBP, and AcGPCR to identify potential protein targets. Prior to the molecular docking process, the protein structures were dehydrated to expose only amino acid residues. Then, polar hydrogens were assigned to the protein structure, nonpolar hydrogens were merged, and Kollman charges were added to amino acid residues. The partial charges and atom types were assigned to stabilized protein structures and saved the files in the PDBQT formats (Protein Data Bank (PDB), Partial Charge (Q), and Atom Type (T)). For the preparation of the ligand, the PubChem database was queried for the 3D structures of pinocembrin (PubChem CID: 68071) ^22^, chrysin (PubChem CID: 5281607) ^23^, and tectochrysin (PubChem CID: 5281954) ^24^. Next, polar hydrogens and Gasteiger charges were introduced to the ligand structures, and nonpolar hydrogens were merged. Finally, the ligand structures were saved in the PDBQT format for stabilized ligand structures. After the receptor and ligand structures were prepared, the grid maps representing the system in the actual docking process were calculated with AutoGrid4 software version 4.2. The dimension of the grid was set to sufficiently cover the whole receptor structure (126 × 126 × 126 Å), with a spacing of 0.608 Å. All procedures were carried out using the AutoDock Auxiliary Tool (ADT) version 4.2 ^25, 26^.

### Molecular docking of Propolis compounds to *Acanthamoeba castellanii* Sir2 family protein, mannose-binding protein, and G-protein coupled receptor

 AutoDock4 version 4.2 ^25, 26^ was chosen for this purpose. Each docking step consisted of 50 GA runs with a maximum population size of 200 units. The total energy evaluation for each docking was 2,500,000 units. The average mutation rate was 0.02, the average cross-over rate was 0.80, and the average elitism value for each docking was 1. The Lamarckian Genetic Algorithm was used to combine local search (using the Solis and Wets algorithm) and global search (using the Genetic Algorithm alone) ^27^. This parameter was used to perform 10,000 independent docking runs on each ligand. This step was repeated five times to ensure the results were accurate. The protein-ligand lowest binding energy (ΔGbind) and the inhibitor constant were determined using AutoDock Auxiliary Tool (ADT) version 4.2 ^25, 26^.

### Molecular dynamics (MD) simulation

MD simulations were performed using the Desmond module (RRID:SCR_014575) from Schrödinger suite (RRID:SCR_014879) ^28^. In this process, hydrogen bonds were assigned according to standard procedures. The optimized potentials for liquid simulations (OPLS) force field was then applied to the protein and ligand complexes. The energy of the complexes was minimized after submerging them in a transferable intermolecular potential with 3 points (TIP3P) water model at a distance of 10 Å from the center of the box. The system was then neutralized by adding sodium and chloride ions, mimicking the *in vivo* environment. Molecular dynamic simulations were performed for 100 ns using ensembles of constant numbers of particles, pressure, and temperature (NPT) with a recording interval of 100 ps. The temperature was set to be 310.15 K and a pressure of roughly 1.01325 bar ^29, 30^.

The following formula was used to determine the root mean square deviation (RMSD) trajectories of the protein-ligand interaction:

$$RMSDx=\sqrt{\frac{1}{N}\sum_{i=1}^{N}(r_{i}^{\prime }(t_{x}))-r_{i}(t_{ref}){)}^{2}}$$

where N is the number of chosen atoms, r' is the position of the chosen atoms in a frame x after they have overlapped in the reference frame, where frame x is captured at time t _*x*,_ and t _*ref*_ is the reference time. Each additional simulation frame required a new repeat of this process ^28^.

The protein residues' root mean square fluctuation (RMSF) trajectories were determined using the following formula:

$$RMSFi=\sqrt{\frac{1}{T}\sum_{t=1}^{T}<(r_{i}^{\prime }(t))-r_{i}(t_{ref}){)}^{2}>}$$

where T stands for the trajectory time interval used to calculate the RMSF, r′ stands for the position of the atoms in residue I following superposition in the reference, r _*i*_ stands for the position of residue I, t _*ref*_ stands for the reference time, and the angle brackets signify that the square distance is averaged on the atoms in the selected residue ^28^.

The ligand atoms' RMSF trajectories were estimated using the following formula:

$$RMSFi=\sqrt{\frac{1}{T}\sum_{t=1}^{T}<(r_{i}^{\prime }(t))-r_{i}(t_{ref}){)}^{2}}$$

where T is the trajectory time interval used to calculate the RMSF, r' is the position of atom I in the reference at time t following superposition on the reference frame, t _*ref*_ is the reference time, and r is the location of atom I in the reference at time t _*ref*_^28^.

Desmond Schrödinger's module's simulation interaction diagram tool was used to analyze protein-ligand interactions, protein-ligand RMSD, and protein and ligand RMSF ^28– 30^.

### Molecular Mechanics Generalized Born Surface Area (MM-GBSA) free energy calculation

The Prime Molecular Mechanics Generalized Born Surface Area (MM-GBSA) approach ^31^, which integrates the GBSA continuum solvent model ^32^, was used to calculate the contributions of enthalpy and entropy-related components toward the binding of the ligand-protein complex. The contributions from molecular mechanics energies, polar solvation, and nonpolar solvation terms were estimated (kcal/mol) using the equation:

$$\Delta {\text{G}}_{\text{bind}}={\text{G}}_{\text{complex}}-{\text{G}}_{\text{protein}}-{\text{G}}_{\text{ligand}}$$

Where,

ΔG _bind_ = Calculated binding free energy of complex

G _complex_ = Binding free energy of minimized complex

G _protein_ = Binding free energy of receptor

G _ligand_ = Binding free energy of unbound ligand

### Protein and ligand visualization

The proteins and ligands in this study were visualized using BIOVIA Discovery Studio version 21.1.0.20298 (RRID:SCR_015651) software ^33^ and the Mol Viewer ^34^.

### Drug likeliness prediction of the ligands using SwissADME analysis

Drug-likeness profiles of ligands were unraveled through SwissADME, a free web tool to evaluate pharmacokinetics, drug-likeness, and medicinal chemistry of small molecules ^35^.

### Pharmacokinetics and toxicity prediction of the ligands

The pharmacokinetic properties of the ligands, such as chemical absorption, distribution, metabolism, excretion, and toxicity (ADMET), were analyzed using the pkCSM ADMET descriptors algorithm methodology, an approach to the prediction of pharmacokinetic properties that relies on graph-based signatures ^36^. In brief, the canonical SMILES of the ligands (pinocembrin, chrysin, and tectochrysin) acquired from the PubChem database (RRID:SCR_004284) were used for input data, and ADMET profiles were generated. The Caco-2 permeability, intestinal absorption (human), and skin permeability were estimated to predict the absorption level of the ligands. The steady-state volume of distribution (VDss), fraction unbound (human), blood-brain barrier (BBB) permeability, and central nervous system (CNS) permeability were evaluated to predict the distribution of the ligands in various tissues. To predict the metabolism of the ligands in the human body, the ligands were determined whether they are likely to be CYP2D6/CYP3A4 substrates (the two main subtypes of cytochrome P450) or Cytochrome P450 inhibitors or not. To predict the excretion of the ligands, total compound clearance was measured. The compounds also determined whether they are likely going to be renal organic cation transporter 2 (OCT2) substrates or not. Finally, the toxicity of the ligands was predicted by AMES toxicity, hERG I/II inhibitor, oral rat acute toxicity (LD _50_), oral rat chronic toxicity (LOAEL), hepatotoxicity, skin sensitization, and Minnow toxicity.

## Results

### Anti- *Acanthamoeba* activities

The effect of Propolis extracts on both strains of *Acanthamoeba* was examined, and the results exhibited as MIC are presented in Table 1 ^37^. Propolis extracts from the Kermanshah city exhibited the most inhibitory activity. The values of the minimum inhibitory concentration (MIC) ranged from 62.5 to 125 µg/mL in trophozoite form. But this extract had no inhibitory activity in cyst form at 1,000 µg/mL concentration. In the positive control, chlorhexidine exhibited MIC values of 8 to 16 and 32 to 64 µg/mL for trophozoite and cyst form, respectively. Therefore, Propolis from Kermanshah city was chosen for further study.

**Table 1.** MIC determination of Propolis extracts and antibiotic against *Acanthamoeba castellanii*.

Propolis extract	MIC value (µg/mL)			
	ATCC 30010		ATCC 50739	
	Trophozoite	Cyst	Trophozoite	Cyst
Sardasht county	>1,000	>1,000	>1,000	>1,000
Boroujen city	>1,000	>1,000	>1,000	>1,000
Kermanshah city	62.5	>1,000	125	>1,000
Chlorhexidine	8	32	16	64

MIC: minimal inhibitory concentration. ATCC: American Type Culture Collection.

### Anti-encystation of *Acanthamoeba castellanii*

**Figure 1.**
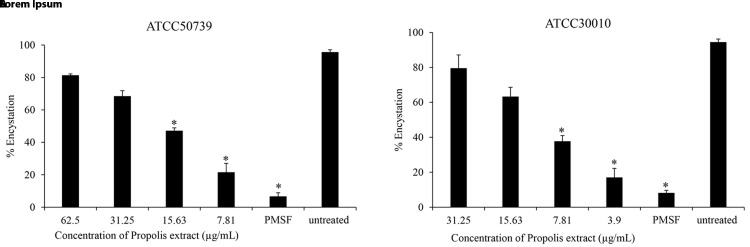
The effect of Propolis extract on encystation. Propolis extract reduced the encystation in a dose-dependent manner on ( **A**) *A. castellanii* ATCC50739 and ( **B**) *A. castellanii* ATCC30010. The experiments were repeated three times, and the average values are presented with error bars representing standard deviations. *; significantly different at a *P* value of <0.05 by Student’s t test. PMSF, phenylmethylsulfonyl fluoride; ATCC, American Type Culture Collection.

To assess the effect of Propolis extract on *A. castellanii* encystation, PMSF was used as a positive control in Neff’s medium. According to the data presented in Figure 1, the results revealed that the Propolis-Kermanshah city exhibited inhibition of *A. castellanii* encystation at all concentrations. The formation of mature cysts significantly reduced after Propolis extract treatment at 1/16 MIC on both strains of *A. castellanii* ATCC50739 (21%) ( Figure 1A) and *A. castellanii* ATCC30010 (17%) ( Figure 1B). In the 5 mM PMSF group, encystation was reduced to 6% and 8% for ATCC50739 and ATCC30010, respectively.

### Anti-excystation of *Acanthamoeba castellanii*

**Figure 2.**
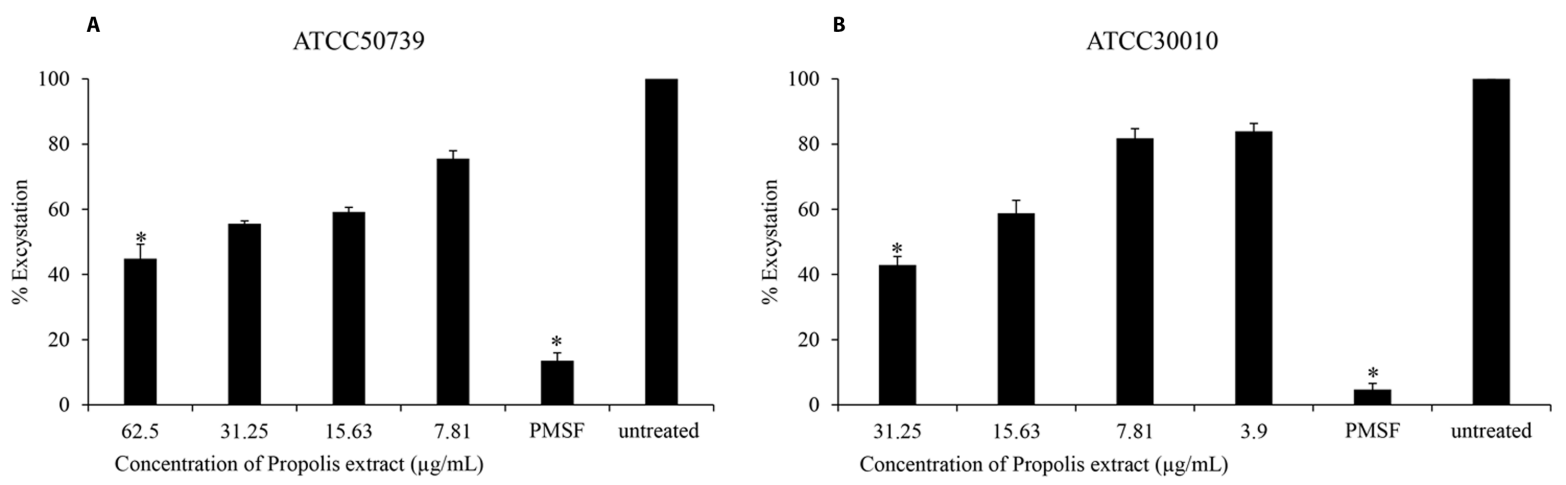
The effect of Propolis extract on excystation. Propolis extract reduced the excystation on ( **A**) *A. castellanii* ATCC50739 and ( **B**) *A. castellanii* ATCC30010. The experiments were repeated three times, and the average values are presented with error bars representing standard deviations. *; significantly different at a *P* value of <0.05 by Student’s t test. PMSF, phenylmethylsulfonyl fluoride; ATCC, American Type Culture Collection.

The effect of Propolis extract treatment on excystation was assessed in PYG medium. The excystation rate decreased to 44% and 42% after exposure to high concentrations of propolis extract at 1/2 MIC of trophozoites ( Figure 2A and B). PMSF significantly inhibited *Acanthamoeba* excystation (13% and 4%) at 5 mM concentration.

### Anti-adhesion assay

To evaluate the influence of Propolis extract on the adhesion properties of *Acanthamoeba* trophozoites, the adhesion of trophozoites to the plastic surface varied and depended on the concentrations of extract. The strongest anti-adhesion was observed in trophozoites treated with 1/2 MIC concentration of extract ( Table 2) ^37^ in both strains of *Acanthamoeba* when compared with the untreated control.

**Table 2.** Effects of Propolis extracts on anti-adhesion of *A. castellanii* ATCC50739 and ATCC30010 trophozoites

Concentration of Propolis extract	Anti-adhesion (%)	
	ATCC50739	ATCC30010
1/2 MIC	55.02 ± 4.14	65.79 ± 3.11
1/4 MIC	41.07 ± 7.53	51.8 ± 0.77
1/8 MIC	17.82 ± 3.68	39.21 ± 3.23
1/16 MIC	13.59 ± 3.13	20.46 ± 2.46
Untreated	100 ± 0	100 ± 0

MIC: minimal inhibitory concentration. ATCC: American Type Culture Collection.

### Toxicity

After 24 hours of treatment with Propolis extract, the number of viable cells was constant at low concentrations, ranging from 8–64 µg/mL. However, the survival rate of Vero cells was lower when treated with the extract at concentrations of 128–1,000 µg/mL.

### GC-MS analysis of Propolis extract

The GC-MS analysis of the Propolis-Kermanshah city extract allowed the identification of 52 compounds. Chrysin (18.86%) was the main compound present in the Propolis extract, followed by pinocembrin (15.02%), and tectochrysin (9.88%), respectively.

### 3D structure prediction of AcSir2, AcMBP, and AcGPCR

**Figure 3.**
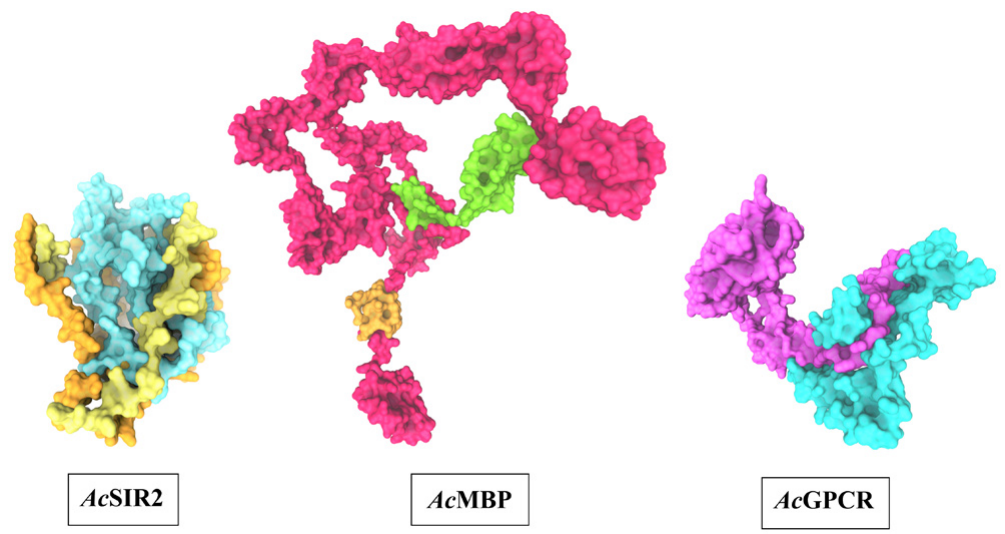
The predicted three-dimensional structures of *Ac*SIR2, *Ac*MBP, and *Ac*GPCR. *Ac*SIR2: blue represents the SIR2 superfamily region, yellow represents the YEATS family region. *Ac*MBP: Orange represents transmembrane proteins; green represents a domain of an unknown function. *Ac*GPCR: purple represents Lung seven-transmembrane receptor. *Ac*SIR2, *A. castellanii* Sir2 family protein; *Ac*MBP, *A. castellanii* mannose-binding protein; *Ac*GPCR, *A. castellanii* G protein-coupled receptor.

The optimal 3D structural models of AcSir2, AcMBP, and AcGPCR were constructed using I-TASSER server and the top 10 threading templates. Then, the best C-score model was selected and refined. The refined 3D structure models of AcSir2, AcMBP, and AcGPCR are illustrated in Figure 3 ^38, 39^. The AcSir2 is a protein located inside the nucleus. The protein consists of 536 amino acids (aa) that contain the SIR2 super-family region (aa residues 36–297) and YEATS family region (aa residues 443-524). The AcMBP is a large protein located at the cell membrane. The protein consists of 833 aa, and some residues such as aa residues 732-760 are transmembrane proteins. The AcGPCR is a protein also located in the cell membrane. The protein consists of 456 aa, and some of them are transmembrane proteins, such as aa residues 182-202, 214-236, 248-274, 286-305, 311-332, 353-375, and 381-401. The protein contains the lung seven-transmembrane receptor region (aa residues 140-413). The stereochemical quality of protein structures was analyzed using PROCHECK. The Ramachandran plot of the AcSir2 model identified 71.0% of the residues in the most favored regions, 25.8% of the residues in the other allowed regions, and only 3.2% of the residues in disallowed regions. The Ramachandran plot of the AcMBP model discovered 64.7% of the residues in the most favored regions, 32.3% of the residues in the other allowed regions, and only 3.0% of the residues in disallowed regions. The AcGPCR model's Ramachandran plot determined 85.5% of the residues in the most favored regions, 13.3% in other allowed regions, and only 1.2% in disallowed regions.

### Molecular docking of Propolis compounds to *A. castellanii* Sir2 family protein, mannose-binding protein, and G-protein coupled receptor

**Figure 4.**
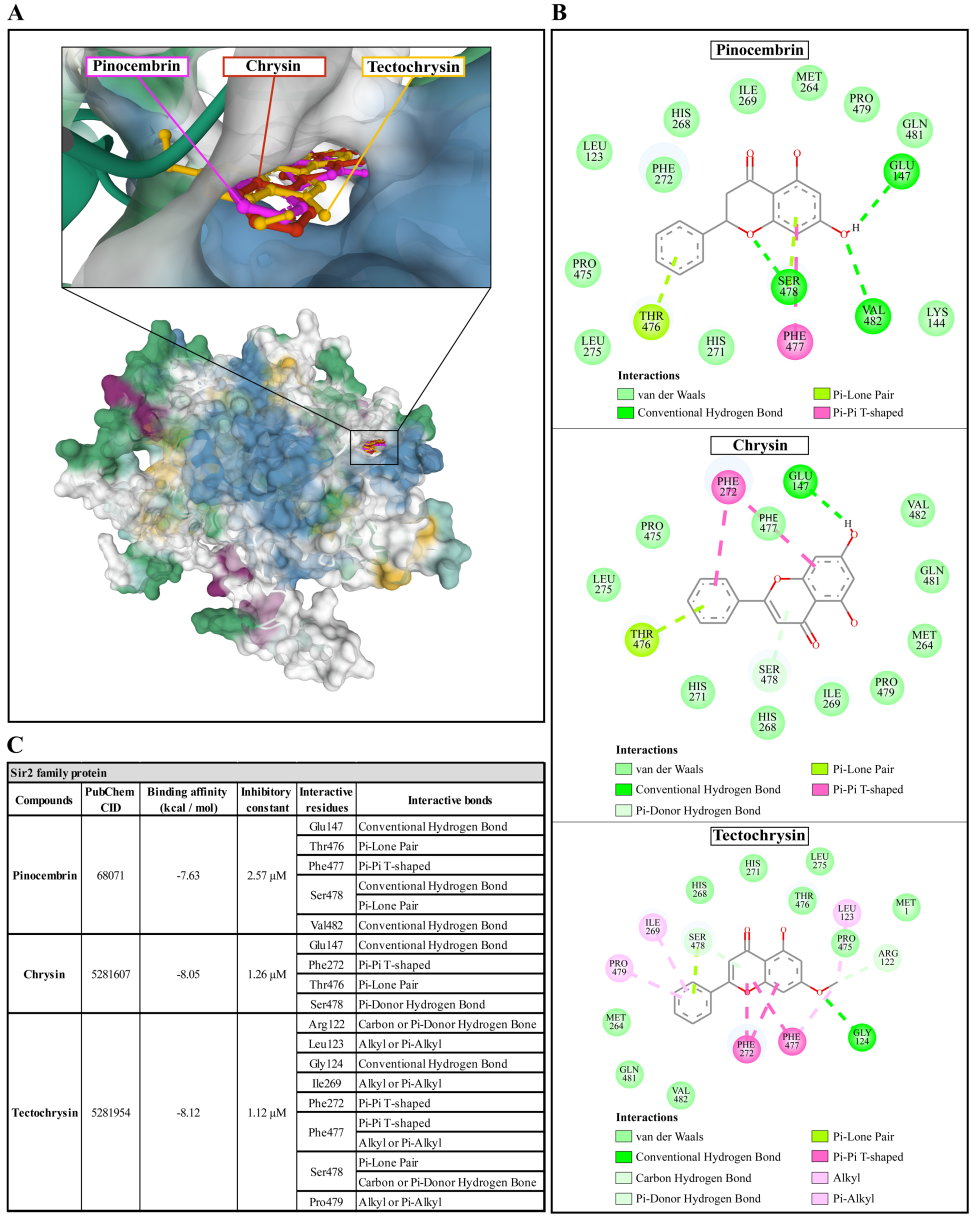
The interaction of pinocembrin, chrysin, and tectochrysin toward the AcSIR2 protein predicted by molecular docking. ( **A**) Binding site of the ligands toward the AcSIR2 protein, purple compound represents pinocembrin, red compound represents chrysin, yellow compound represents tectochrysin. ( **B**) A schematic representation of the detailed interactions of the ligand atoms with the protein residues. ( **C**) Binding affinity and inhibitory constant prediction of propolis compounds against Sir2 family protein of *Acanthamoeba castellanii*. AcSIR2, *A. castellanii* Sir2 family protein.

The molecular docking of Propolis compounds such as: pinocembrin, chrysin, and tectochrysin against three essential proteins of *A. castellanii* was performed using AutoDock 4. The results are illustrated in Figure 4– Figure 6 ^39, 40^. The Pinocembrin demonstrated good binding potential to the AcSir2 with binding energy (ΔGbind) of -7.63 kcal/mol and the inhibitory constant (Ki) of 2.57 µM. The compound interacts with the residues Glu147 through the conventional hydrogen bond (H-bond), Thr476 through Pi-lone pair, Phe477 through Pi-Pi T-shaped, Ser478 through conventional H-bond and Pi-Lone Pair, and Val482 through conventional H-bond. Chrysin exhibited a high affinity for AcSir2, with a ΔGbind of -8.05 kcal/mol and a Ki of 1.26 µM. The compound interacts with the residues Glu147 *via* the conventional H-bond, Phe272 *via* Pi-Pi T-shaped, Thr476 *via* Pi-lone pair, and Ser478 *via* Pi-donor H-bond. Tectochrysin exhibited an excellent affinity for AcSir2, with a ΔGbind of -8.12 kcal/mol and a Ki of 1.12 µM. The compound interacts with residues Arg122 through carbon or Pi-donor H-bond, Leu123 through alkyl or Pi-alkyl, Gly124 through conventional H-bond, Ile269 through alkyl or Pi-alkyl, Phe272 through Pi-Pi T-shaped, Phe477 through Pi-Pi T-shaped, and alkyl or Pi-alkyl, Ser478 through Pi-lone pair, and carbon or Pi-donor H-bone, and Pro479 through alkyl or Pi-alkyl ( Figure 4).

**Figure 5.**
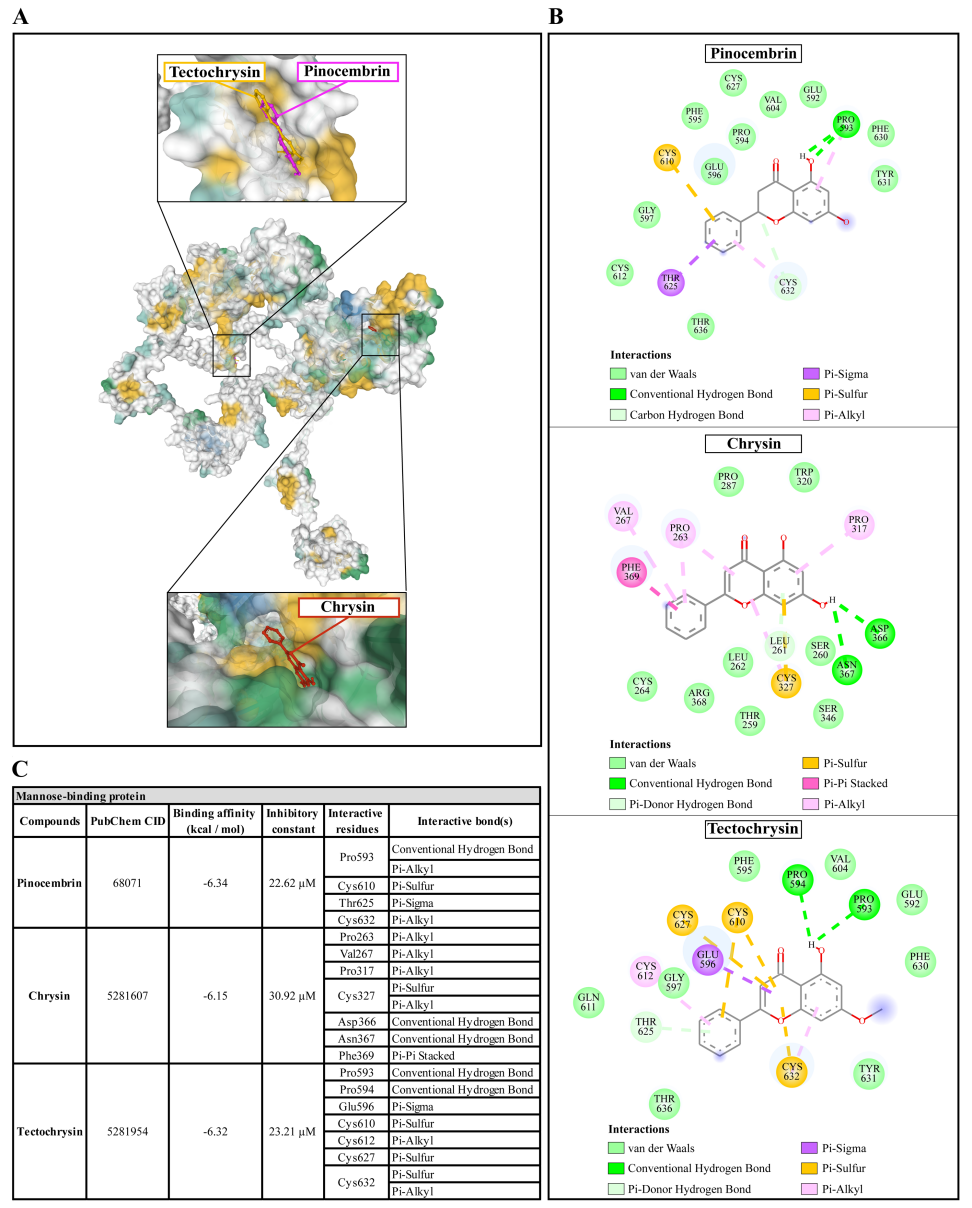
The interaction of pinocembrin, chrysin, and tectochrysin toward the AcMBP protein predicted by molecular docking. ( **A**) Binding site of the ligands toward the AcMBP protein, purple compound represents pinocembrin, red compound represents chrysin, yellow compound represents tectochrysin. ( **B**) A schematic representation of the detailed interactions of the ligand atoms with the protein residues. ( **C**) Binding affinity and inhibitory constant prediction of propolis compounds against mannose-binding protein of *Acanthamoeba castellanii*. AcMBP, *A. castellanii* mannose-binding protein.

**Figure 6.**
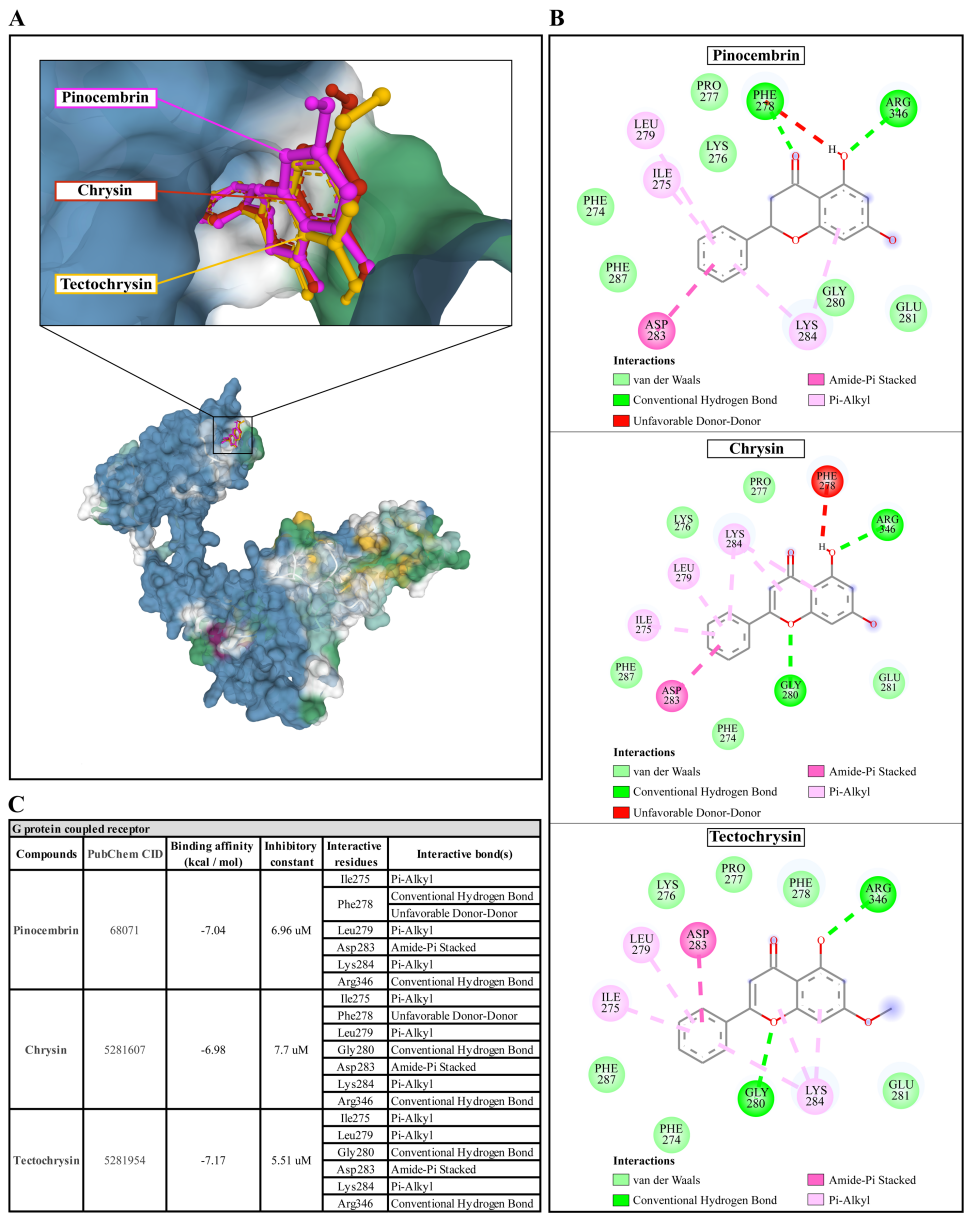
The interaction of pinocembrin, chrysin, and tectochrysin toward the AcGPCR protein predicted by molecular docking. ( **A**) Binding site of the ligands toward the AcSIR2 protein, purple compound represents Pinocembrin, red compound represents Chrysin, yellow compound represents Tectochrysin. ( **B**) A schematic representation of the detailed interactions of the ligand atoms with the protein residues. ( **C**) Binding affinity and inhibitory constant prediction of propolis compounds against G protein-coupled receptor of *Acanthamoeba castellanii*. AcGPCR, *A. castellanii* G protein-coupled receptor; AcSIR2, *A. castellanii* Sir2 family protein.

Pinocembrin demonstrated a weak binding affinity for AcMBP with ΔGbind of -6.34 kcal/mol and the Ki of 22.62 µM. The compound interacts with residues Pro593 through conventional H-bond and Pi-alkyl, Cys610 through Pi-sulfur, Thr625 through Pi-sigma, and Cys632 through Pi-alkyl. Chrysin had a very low affinity for AcMBP, with ΔGbind of -6.15 kcal/mol and the Ki of 30.92 µM. The compound interacts with the residues Pro263, Val267, and Pro317 through Pi-alkyl; Cys327 through Pi-sulfur and Pi-alkyl; Asp366 and Asn367 through conventional H-bond; and Phe369 through Pi-Pi Stacked. Tectochrysin showed a low affinity for AcMBP, with ΔGbind of -6.32 kcal/mol and the Ki of 23.21 µM. The compound interacts with residues Pro593 and Pro594 through Conventional H-bond; Glu596 through Pi-sigma; Cys610, Cys627, and Cys632 through Pi-sulfur; and Cys612 and Cys632 through Pi-Alkyl ( Figure 5).

Pinocembrin demonstrated a weak binding affinity for the AcGPCR with ΔGbind of -7.04 kcal/mol and the Ki of 6.96 µM. The compound interacts with residues Ile275 through Pi-alkyl, Phe278 through conventional H-bond and unfavorable donor-donor interaction, Leu279 through Pi-alkyl, Asp283 through Amide-Pi stacked, Lys284 through Pi-alkyl, and Arg346 through conventional H-bond. With a ΔGbind of -6.98 kcal/mol and Ki of 7.7 µM, chrysin exhibited a low affinity for AcGPCR. The compound interacts with AcGPCR in the same way as the pinocembrin-AcGPCR complex, except for the residue Phe278, whose conventional H-bonding did not occur for this compound. Finally, tectochrysin exhibited a low affinity for AcGPCR, with ΔGbind of -7.17 kcal/mol and the Ki of 5.51 µM. The compound interacts with AcGPCR the same as the pinocembrin-AcGPCR complex, except for the residue Phe278 in which there was no interaction for this compound ( Figure 6). Based on the molecular docking result, pinocembrin, chrysin, and tectochrysin demonstrated inhibition potential towards the AcSir2 protein. Tectochrysin showed the most robust inhibition, followed by chrysin and pinocembrin. Thus, the molecular dynamics of these complexes were then simulated to understand the dynamic motions and analyze the stabilities of these protein-ligand complexes.

### Molecular dynamic simulations of apo and bound forms of AcSir2 protein

**Figure 7.**
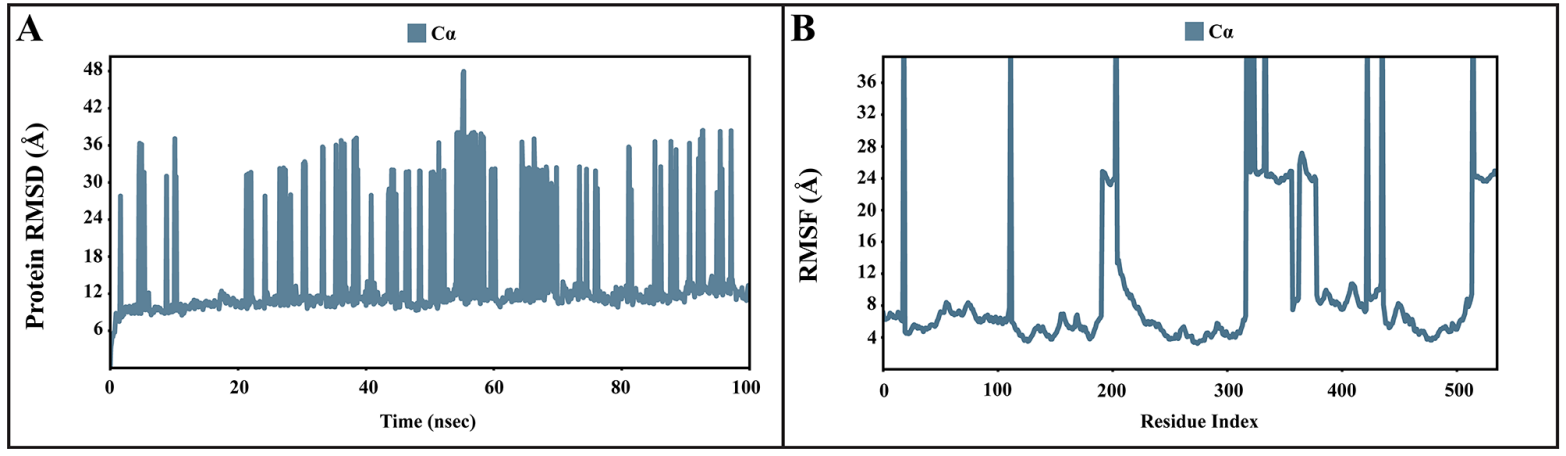
The P-RMSD and P-RMSF of the apo-AcSIR2 protein. ( **A**) Plot of the P-RMSD of the apo-AcSIR2 protein. ( **B**) Plot of the P-RMSF of the apo-AcSIR2 protein. P-RMSD, protein root mean square deviation; P-RMSF, protein root mean square fluctuation; AcSIR2, *A. castellanii* Sir2 family protein.

**Figure 8.**
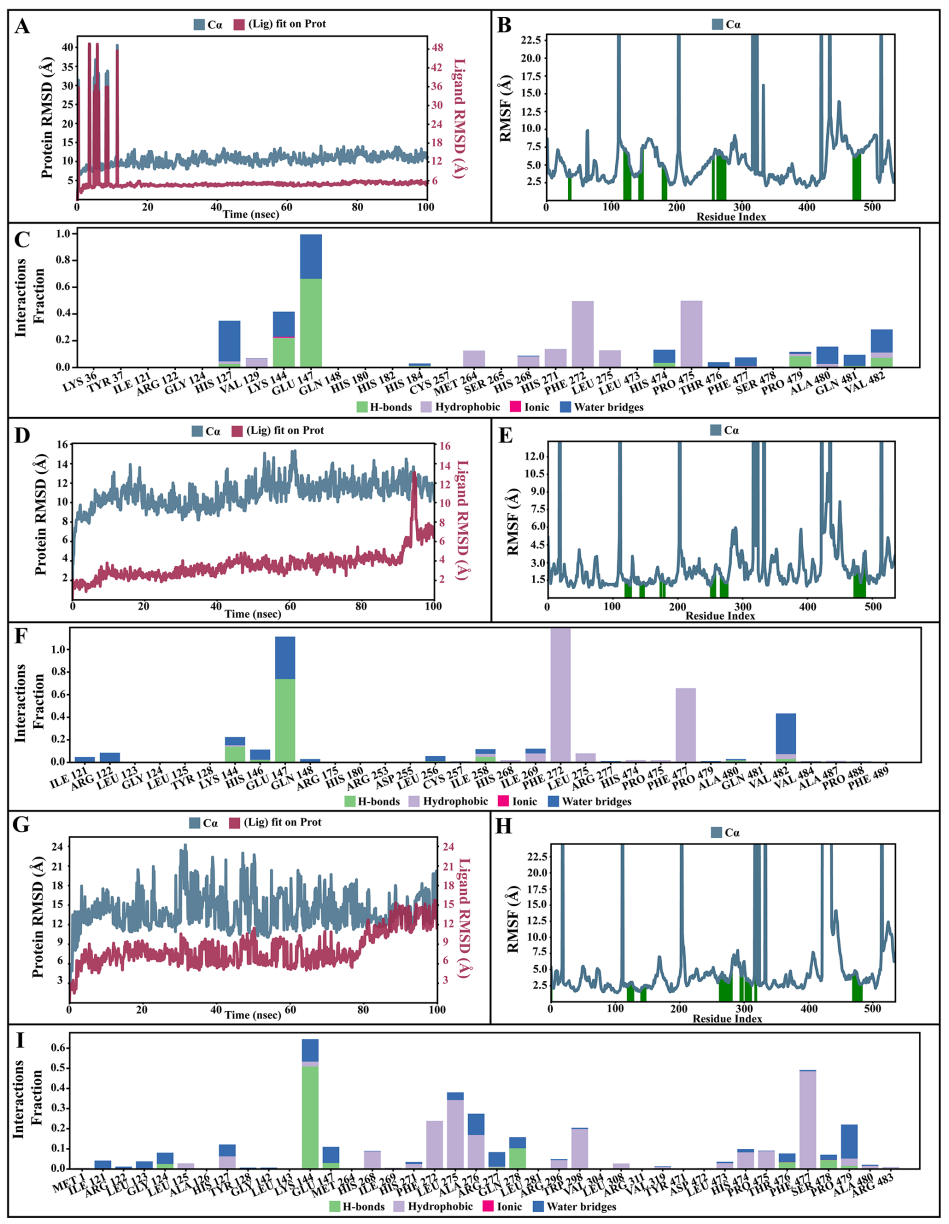
Simulation interactions diagram of pinocembrin, chrysin and tectochrysin with essential proteins. ( **A–C**) Simulation interactions diagram of pinocembrin-AcSIR2 complex. (A) Plot of protein-ligand RMSD. ( **B**) Plot of protein RMSF. ( **C**) Histogram of protein-ligand contacts categorized by type of interactions: hydrogen bonds (green), hydrophobic (purple), ionic (magenta), and water bridges (blue). (D-F) Simulation interactions diagram of chrysin-AcSIR2 complex. ( **D**) Plot of protein-ligand RMSD. ( **E**) Plot of protein RMSF. ( **F**) Histogram of protein-ligand contacts categorized by type of interactions: hydrogen bonds (green), hydrophobic (purple), ionic (magenta), and water bridges (blue). ( **G–I**) Simulation interactions diagram of tectochrysin-AcSIR2 complex. ( **G**) Plot of protein-ligand RMSD. (H) Plot of protein RMSF. ( **I**) Histogram of protein-ligand contacts categorized by type of interactions: hydrogen bonds (green), hydrophobic (purple), ionic (magenta), and water bridges (blue). RMSD, root mean square deviation; RMSF, root mean square fluctuation; AcSIR2, *A. castellanii* Sir2 family protein.

The dynamic motions of the apo and docked complexes were further analyzed by molecular dynamic simulations at 100 ns using the Desmond module of Schrödinger's suite. The results of MD simulations of the apo and bound forms of AcSir2 protein are illustrated in Figure 7 and Figure 8 ^39, 41^, respectively. For an MD run of 100 ns, the RMSD and the RMSF were predicted for the apo and bound forms. A ligand’s interaction can ward off unfolding and stabilize the protein ^42^. Hence, we analyzed the protein’s secondary structures before and after docking to understand the conformational changes due to ligand binding. The RMSD quantifies the average change in displacement of a selection of atoms relative to a reference frame for a particular frame. Figure 7A, Figure 8A, 8D, and 8G demonstrated the protein RMSD from the simulation of the AcSir2 apo form, pinocembrin-AcSir2 complex, chrysin-AcSir2 complex, and tectochrysin-AcSir2 complex, respectively. The Protein RMSD (P-RMSD) shows how the RMSD of a protein has changed over time (left Y-axis). After aligning all of the protein frames with the backbone of the reference frame, the atoms are chosen to figure out the P-RMSD. During the simulation, the calculation of the P-RMSD can give information about how the structure is built. For the ligand RMSD (L-RMSD), the L-RMSD value (right Y-axis) shows how stable the ligand is concerning the protein and its binding pocket. 'Lig fit Prot' illustrated the RMSD of a ligand after the protein-ligand complex was aligned in the reference protein backbone and the RMSD of the ligand heavy atoms was determined. If the observed values exceed the P-RMSD by a significant amount, the ligand almost certainly has diffused away from its initial binding site. The mean values of P-RMSD of the apo-AcSir2 was around the 10 Å ( Figure 7A). The P-RMSD values of the Pinocembrin-AcSir2 complex wildly deviated at around the first 12 ns. After that, the fluctuation was regular at the end of the simulation. An average of RMSD values is stable after 12 ns, indicating that the system has equilibrated during this simulation. Furthermore, for the L-RMSD values of the Pinocembrin-AcSir2 complex, the observed values are significantly lower than the P-RMSD, so the ligand has likely fixed in its initial binding site ( Figure 8A, Supplementary video 1 found as *Underlying data* ^43^). The P-RMSD values of the chrysin-AcSir2 complex deviated from 0 to 14 Å in the first duration of 18 ns and slightly decreased during 18 ns to 50 ns. The fluctuation was regular at the end of the simulation after 70 ns, with the highest point about 15 Å and the lowest point about 10 Å. An average of RMSD values is constant after 70 ns, indicating that the system has equilibrated during this simulation. The L-RMSD values of this complex show significantly lower than the RMSD of the protein during the first 90 ns. However, the values dramatically increase around 90 ns and are higher than the P-RMSD. Almost obviously, the ligand may have diffused away from its initial binding site ( Figure 8D, Supplementary video 2 found as *Underlying data* ^43^). The fact that the P-RMSD values of the tectochrysin-AcSir2 complex strongly deviated throughout the simulation shows that a substantial conformational change has occurred in the protein. However, the overall average is relatively stable after 60 ns, indicating that the system has equilibrated during this simulation. The L-RMSD values of this complex are lower than the RMSD of the protein during the first 80 ns. However, the values gradually increase during the simulation time of 80–90 ns, then more extensive than the P-RMSD at approximately 90 ns. Clearly, the ligand may have diffused away from its initial binding site ( Figure 8G, Supplementary video 3 found as *Underlying data* ^43^). The RMSF can characterize local changes in the protein chain and the positions of the ligand atoms. Figure 7B, Figure 8B, 8E, and 8H demonstrated the protein and ligand RMSF from the simulation of the apo-AcSir2, pinocembrin-AcSir2 complex, chrysin-AcSir2 complex, and tectochrysin-AcSir2 complex, respectively. For the protein RMSF (P-RMSF), the peaks in this plot correspond to the protein regions that fluctuate the most during the simulation. The P-RMSF of the apo-AcSir2 strongly fluctuated at amino acid residues Pro19, Pro112, Cys192-Gly212, Pro318-Ala357, Arg364-Met378, Thr409-Glu412, Pro423, His432, Ala434-Pro436, and Pro515-Ala536 ( Figure 7B). The P-RMSF of the pinocembrin-AcSir2 complex strongly fluctuated at amino acid residues Pro112, Pro204, Asp317, Pro320, Pro324, Pro334, Pro423, Pro436, Val450, and Pro515. However, these residues were not ligand contacts residues ( Figure 8B). The P-RMSF of the chrysin-AcSir2 complex immensely fluctuated at amino acid residues Pro19, Pro112, Pro204, Asp317, Pro324, Pro334, Pro423, His432, Pro436, Lys451, and Pro515. These residues were also not ligand contacts residues ( Figure 8E). The P-RMSF of the tectochrysin-AcSir2 complex wildly fluctuated at amino acid residues Pro19, Pro112, Pro204, Val319, Pro324, Pro423-Thr437, Thr443, Pro151, and Gly525. Almost all these residues were not ligand contacts residues, except for the Pro204 position ( Figure 8H). The result illustrated that the binding of a ligand can prevent protein unfolding and stabilize it. During the simulation, the interactions of the protein with the ligand can be monitored. The protein-ligand contacts diagrams for the pinocembrin-AcSir2 complex, chrysin-AcSir2 complex and tectochrysin-AcSir2 complex are illustrated in Figures 8C, 8F, and 8I, respectively. The stacked bar charts demonstrated that all complexes exhibited H-bonds, hydrophobic interactions, ionic bonds, and water bridges during the simulation. The PRIME MM-GBSA binding free energy values of the ligand-AcSir2 complexes are given in Table 3.

**Table 3.** Prime MM-GBSA binding free energy values (kcal/mol) of the ligand-protein complexes.

Complex	^1^MM-GBSA ΔBind	^2^MM-GBSA ΔCoul	^3^MM-GBSA ΔHbond	^4^MM-GBSA ΔLipo	^5^MM-GBSA ΔvdW
Pinocembrin-AcSir2 complex	-64.376	-35.280	-11.711	-27.255	-75.361
Chrysin-AcSir2 complex	-111.971	-161.417	-21.694	-31.961	-65.150
Tectochrysin-AcSir2 complex	-108.284	-127.562	-20.367	-27.298	-84.088

^1^Binding free energy, ^2^Coulombic energy, ^3^Hydrogen bond energy, ^4^Lipophilic energy, ^5^Van der Waal energy. MM-GBSA: Molecular Mechanics Generalized Born Surface Area. AcSIR2: *A. castellanii* Sir2 family protein.

### Drug likeliness prediction of the ligands using SwissADME

After careful analysis of the drug-likeness properties of the ligands using SwissADME, the result indicated that the compound properties are within the range of drug-likeness based on various filters such as Lipinski ^44^, Ghose ^45^, Veber ^46^, Egan ^47^, and Muegge ^48^ ( Table 4).

**Table 4.** Drug likeliness prediction of the ligands using SwissADME.

Drug likeness			
Filters	Pinocembrin	Chrysin	Tectochrysin
Lipinski	Yes; 0 violation	Yes; 0 violation	Yes; 0 violation
Ghose	Yes	Yes	Yes
Veber	Yes	Yes	Yes
Egan	Yes	Yes	Yes
Muegge	Yes	Yes	Yes
Bioavailability Score	0.55	0.55	0.55

### Pharmacokinetics and toxicity prediction of the ligands

The ADMET properties of the pinocembrin, chrysin, and tectochrysin are presented in the Table 5. To predict the absorption level of the compounds, water solubility, Caco-2 permeability, intestinal absorption (human), and skin permeability were estimated. A compound is easy to absorb if Caco-2 permeability is high. The Caco-2 permeability is considered as high if it has an apparent permeability coefficient (Papp) > 8 × 10 ^-6^ cm/s (or log Papp > 0.90). The results showed that all ligands were predicted to have high Caco-2 permeability. About the human intestinal absorption prediction, a compound is poorly absorbed if absorbance is less than 30%. The results proved that all compounds were considered to have a good absorption. With regards to skin permeability, if a compound has a logKp > -2.5, the compound is predicted to have a relatively low skin permeability. The results indicated that all compounds were predicted to have good skin permeability.

**Table 5.** Pharmacokinetics and toxicity prediction of the ligands.

Property	Predicted Value			Unit
	Pinocembrin	Chrysin	Tectochrysin	
	68071	5281607	5281954	
**Absorption**				
Water solubility	-3.538	-3.538	-3.641	log mol/L
CaCo _2_ permeability	1.152	0.945	1.248	log Papp in 10-6 cm/s
Intestinal absorption (human)	92.417	93.761	95.229	% Absorbed
Skin Permeability	-2.808	-2.739	-2.758	log Kp
P-glycoprotein substrate	Yes	Yes	Yes	Yes/No
P-glycoprotein I inhibitor	No	No	No	Yes/No
P-glycoprotein II inhibitor	No	No	Yes	Yes/No
**Distribution**				
VDss (human)	-0.386	0.403	-0.047	log L/kg
Fraction unbound (human)	0.022	0.136	0.119	Fu
BBB permeability	0.42 (readily cross the BBB)	0.047	0.003	log BB
CNS permeability	-2.047	-1.912 (penetrate the CNS)	-1.992 (penetrate the CNS)	log PS
**Metabolism**				
CYP2D6 substrate	No	No	No	Yes/No
CYP3A4 substrate	No	No	Yes	Yes/No
CYP1A2 inhibitor	Yes	Yes	Yes	Yes/No
CYP2C19 inhibitor	Yes	Yes	Yes	Yes/No
CYP2C9 inhibitor	Yes	Yes	Yes	Yes/No
CYP2D6 inhibitor	No	No	No	Yes/No
CYP3A4 inhibitor	No	No	Yes	Yes/No
**Excretion**				
Total Clearance	0.122	0.405	0.457	log mL/min/kg
Renal OCT2 substrate	No	No	No	Yes/No
**Toxicity**				
AMES toxicity	No	No	No	Yes/No
hERG I inhibitor	No	No	No	Yes/No
hERG II inhibitor	No	No	No	Yes/No
Oral rat acute toxicity (LD _50_)	1.586	2.289	2.042	mol/kg
Oral rat chronic toxicity	2.059	0.955	0.744	log mg/kg- bw/day
Hepatotoxicity	No	No	No	Yes/No
Skin sensitization	No	No	No	Yes/No
Minnow toxicity	1.683	1.746	-0.04	log mM

VDss: the steady state volume of distribution. BBB: blood-brain barrier. CNS: central nervous system. AMES: assay of the ability of a chemical compound to induce mutations in DNA. OCT2: organic cation transporter 2.

To predict the distribution of the compounds in various tissues, the VDss, Fraction unbound (human), BBB permeability, and central CNS permeability were evaluated. The VDss is relatively low if lower than 0.71 L/kg (log VDss < -0.15). Whereas it is high if higher than 2.81 L/kg (log VDss > 0.45). The results demonstrated that the VDss of the pinocembrin was higher than the chrysin and tectochrysin. The higher the VD is, the more of a ligand is distributed in tissue rather than plasma, as demonstrated in the pinocembrin. With regards to BBB and CNS permeability, the results indicated that the pinocembrin readily crossed the BBB, while chrysin and tectochrysin might penetrate the CNS.

As cytochrome P450 is responsible for the metabolism of many drugs in liver, to predict metabolism of compounds, the compounds were determined whether they are likely to be CYP2D6/CYP3A4 substrates (the two main subtypes of cytochrome P450) or Cytochrome P450 inhibitors or not. The result predicted that all compounds were not substrates and inhibitors of the CYP2D6. However, all of them were inhibitors of CYP1A2, CYP2C19, and CYP2C9. Moreover, the tectochrysin was a substrate and inhibitor of CYP3A4.

To predict the excretion of the compounds, total compounds clearance was measured. It was also determined whether the compounds were likely going to be renal organic cation transporter 2 (OCT2) substrates or not. With regards to total compounds clearance, the total clearance of the tectochrysin is the highest, followed by chrysin, and pinocembrin. Remarkably, all compounds were not predicted to be renal OCT2 substrates.

Finally, to predict the toxicity of the compounds, AMES toxicity, hERG I/II inhibitor, oral rat acute toxicity (LD _50_), oral rat chronic toxicity (LOAEL), hepatotoxicity, skin sensitization, and Minnow toxicity were predicted. Notably, the results predicted that none of the compounds were mutagenic or hERG I/II inhibitors, and none of them showed hepatotoxicity or skin sensitization.

## Discussion

The pharmaceutical activities of natural products have historically been screened because they are thought to have key roles in drug discovery, are inexpensive and rarely have undesirable side effects. Propolis has been known for a long time and attracted scientific interest due to its biological activities such as anti-viral, anti-bacterial, anti-fungal, anti-protozoal, anesthetic, antioxidant, anti-tumoral, anti-cancer, anti-hepatotoxic, anti-mutagenic, anti-septic and anti-inflammatory activities, in addition to being utilized for its cytotoxic activity ^49, 50^. *In vitro* studies of its antiparasitic effect were reported against *Leishmania* spp., *Trypanosoma* spp., *Plasmodium* spp., *Cryptosporidium* spp., *Giardia* spp., *Toxoplasma gondii*, *Trichomonas vaginalis*, and *Blastocystis* spp. ^51^. In literature, Propolis extract has reported amoebistatic activity between 2.0 and 6.0 mg/mL and its effects were amoebicidal at 8.0 mg/mL or higher ^52^. Here, the anti- *Acanthamoeba* activities of three Propolis extracts from different cities in Iran were screened. The highest activity was obtained from the Propolis ethanolic extract of Kermanshah city, and the MIC against trophozoites was 62.5 µg/mL and 125 µg/mL for *A. castellanii* ATCC30010 and ATCC50739, respectively. Propolis composition included more than 180 different types of chemicals ^53^ depending on several factors such as extraction method, source of plant, season, and local flora ^54^. Flavonoid compounds like chrysin and pinocembrin are commonly identified in Romanian, Turkish and Polish propolis. The type of Uruguayan propolis mentioned other flavonoid compounds like tectochrysin, galangin and kaempferol ^55^. This study revealed the main compounds of Propolis from Kermanshah city were chrysin, pinocembrin, and tectochrysin. To determine the cytotoxic effect of the extract at a concentration of at least 0.128 mg/mL was demonstrated against Vero cells. Our data agreed with Vural *et al.* ^56^, in which the Propolis concentration at higher than 7.81 mg/mL caused corneal epithelial cell damage. The safe concentration of Propolis at 1.4 mg/kg per day was also recommended ^57^.

*A. castellanii* ATCC50739 and ATCC30010 were tested for their encystment capability in Neff’s medium. The results seemed to encyst and presented the mature cysts in both media for seven days. The process of *Acanthamoeba* is an essential for the survival under unfavorable conditions ^58^. The double wall of the *Acanthamoeba* cyst is resistant to many drugs and chemicals and leads to clinical drug resistance ^59^. As only single cyst surviving in the cornea stroma after initial successful treatment, they can regularly excyst and lead to reinfection ^60^. Thus, the inhibition of encystation process during the treatment of *Acanthamoeba* infections can lead to more favorable outcomes and enhances the potential of *Acanthamoeba* keratitis treatment. In this study, PMSF was used as a positive control to block serine proteinase, providing a significant inhibition of encystation. The data were in agreement with the results of Leitsch *et al.* ^61^ in which the PMSF inhibited the proteolytic activity at the early stage of encystation. Propolis extract at low concentration (1/16 MIC) was able to inhibit the encystation of *A. castellanii* ATCC50739 and ATCC30010. The low concentration caused a reduction in the level of encystation of around 80–90%. The high concentrations (1/2-1/8 MIC) gave a < 20% reduction in the encystment levels, which suggests that low concentration of Propolis extract is suitable for inhibiting the encystment process. The main mechanism underlying inhibition of encystation by Propolis remain largely unknown. Aqeel *et al.* ^62^ mentioned phenolic compounds such as resveratrol and demethoxycurcumin are strong antioxidants with *Acanthamoeba* growth inhibitory effects *in vitro*. It raises the possibility that antioxidant activity may be required to inhibit *Acanthamoeba* encystation. Furthermore, Mahboob *et al.* ^63^ reported that other phenolic compounds *i.e.*, ester of caffeic acid and quinic acid, demonstrated the inhibitory effect on encystation by scavenging reactive oxygen species within *Acanthamoeba* cytoplasm.

The use of therapeutic agents for *Acanthamoeba* infection may lead to cyst formation, a drug-resistance stage, and transformation of cysts to trophozoites that lead to recurrence of infection ^64^. The fluids or some microorganisms in eye infection may provide an appropriate condition to induce excystation of surviving *Acanthamoeba* cyst ^65^. This reason remains a challenge for *Acanthamoeba* keratitis prevention. Although Propolis extract from Kermanshah city did not inhibit the growth of *Acanthamoeba* cysts at 1,000 µg/mL, it exhibited excystment inhibition at 62.5 and 31.25 µg/mL. This evidenced that it prevented the recurrence of infection because there was no change of morphological transformation from cysts to trophozoites. However, it remains unclear on how Propolis inhibited *Acanthamoeba* excystation. Maslinic acid, a natural triterpene found in olives and Propolis, has been shown to inhibit parasitic proteases enzymes ^66^. These proteases enzymes are normally secreted within the first 24 hours, which may indicate an important role of the enzyme in excystation ^67^.

The first step in the pathogenesis of *Acanthamoeba* infection is the adhesion to the surface of the host tissues. Subsequently, the adhesion to host cells, *Acanthamoeba* produce proteinase enzymes that work in concert to produce a potent cytopathic effect (CPE) involving killing of the host cells, degradation of epithelial basement, and penetration into the deeper layers of the cornea ^68^. In this study, we showed that Propolis possess anti-amoebic properties and the capability to reduce amoebae adhesion on plastic plate. The highest adhesion was noticed in the control group, which was an untreated agent. Similar results were obtained in the current study, where anti-adhesion was observed in plastic plate and contact lenes belonging to *Curcuma longa* extract ^69^, *Annona muricata* and *Combretum trifoliatum* extracts ^70^, and *Garcinia mangostana* and their pure compounds ^13^.

Based on our results, we recognize the importance of developing Propolis extract to eliminate or inhibit the pathogenicity of *Acanthamoeba*. Therefore, the determination the main target of the pathogen *in silico* has been studied. A molecular docking simulation was carried out to investigate the binding affinities of the major compounds (chrysin, pinocembrin and tectochrysin) from the Propolis extract and essential proteins in *Acanthamoeba* (AcSir2, AcMBP, and AcGPCR). AcSir2 was classified as a class-IV sirtuin. This protein exhibited functional SIRT deacetylase activity, localized mainly in the nucleus, and its transcription was upregulated during encystation ^71^. *Acanthamoeba* mannose-binding protein (AcMBP) is a virulence factor of the free-living amoeba, which is important for adhesion of the pathogen ^72^. G proteins and GPCRs are well known key regulators of cellular communication and cellular functions including cell cycle, mitosis, and proliferation ^73^.

Our study revealed the potential capability of the pinocembrin, chrysin, and tectochrysin complex to form hydrogen bonds with the AcSir2 protein. The low binding energy indicates strong interactions between the compounds and AcSir2 protein. Sirtuins have been classified into five major classes (I, II, III, IV and V) and conserved from bacteria to humans ^71^. In some parasites, Sir2 is located mainly in the nucleus and plays a role in cell function, proliferative life span and development under various conditions ^74^. Notably, the regulation of AcSir2 expression is essential for growth and encystation in *A. castellanii*. In AcSir2-overexpressing encysting cells, the transcription of cellulose synthase was highly upregulated compared to control cells ^71^. Moreover, MD simulations indicated that pinocembrin, chrysin, and tectochrysin can interact with AcSir2 protein. Chrysin and tectochrysin may have a probability of diffusing away from their initial binding site. Over the 100 ns of MD simulations, only the pinocembrin remained fixed within its initial binding site. Thus, this compound may be an excellent candidate for future anti- *Acanthamoeba* drug development. In this study, our successful combination of computational approaches and phenotypic screening led to the identification of compounds with noteworthy activities against *Acanthamoeba*.

## Conclusions

Natural products are one of the essential resources for drug discovery. Considering the pharmacological activities of Propolis extract against *Acanthamoeba*, its therapeutic potential should be considered. Our study was conducted with extract of Propolis. Moreover, molecular docking was used as a computational and easily accessible method to propose a binding mode of chrysin, tectochrysin and pinocembrin on a protein target. Molecular docking stimulation indicated that pinocembrin is the strongest binding site on AcSir2 protein. This noteworthy data further allows us to simulate the effects of pinocembrin or its synthetic structural modifications to optimize desirable activities and targets. Nevertheless, our results provide the possibility of finding a new series of anti- *Acanthamoeba* compounds that can act in combination with conventional drugs as an alternative therapeutic strategy for the treatment of AK.

## Data Availability

NCBI Protein **:** transcriptional regulator, Sir2 family protein [ *Acanthamoeba castellanii* str. Neff]. Accession number XP_004358245.1; https://identifiers.org/ncbiprotein:xp004358245.1 ^18^. NCBI Protein: mannose-binding protein [ *Acanthamoeba castellanii*]. Accession number AAT37865.1; https://identifiers.org/ncbiprotein:AAT37865.1 ^19^. NCBI Protein: G protein coupled receptor, putative [ *Acanthamoeba castellanii* str. Neff]. Accession number ELR16814.1; https://identifiers.org/ncbiprotein:ELR16814.1 ^18^. NCBI PubChem Compound: Pinocembrin. PubChem CID 68071; https://identifiers.org/pubchem.compound:68071 ^22^. NCBI PubChem Compound: Chrysin. PubChem CID 5281607; https://identifiers.org/pubchem.compound:5281607 ^23^. NCBI PubChem Compound: Tectochrysin. PubChem CID 5281954; https://identifiers.org/pubchem.compound:5281954 ^24^. Figshare: *In vitro* RAW DATA.xlsx. https://doi.org/10.6084/m9.figshare.21213563 ^37^. Figshare: MD simulations Movie.rar. https://doi.org/10.6084/m9.figshare.21213560 ^43^. This project contains the following underlying data: Supplementary videos 1-3 Figshare: Raw data for Molecular docking. https://doi.org/10.6084/m9.figshare.21214079 ^40^. This project contains the following underlying data: Molecular docking result between Propolis compounds and *A. castellanii* G-protein coupled receptor (AcGPCR) Pinocembrin (PubChem CID: 68071) docking with AcGPCR Chrysin (PubChem CID: 5281607) docking with AcGPCR Tectochrysin (PubChem CID: 5281954) docking with AcGPCR Molecular docking result between Propolis compounds and *A. castellanii* mannose-binding protein (AcMBP) Pinocembrin (PubChem CID: 68071) docking with AcMBP Chrysin (PubChem CID: 5281607) docking with AcMBP Tectochrysin (PubChem CID: 5281954) docking with AcMBP Molecular docking result between Propolis compounds and *A. castellanii* Sir2 family protein (AcSir2) Pinocembrin (PubChem CID: 68071) docking with AcSir2 Chrysin (PubChem CID: 5281607) docking with AcSir2 Tectochrysin (PubChem CID: 5281954) docking with AcSir2 Figshare: Raw data for Molecular dynamics (MD) simulation. https://doi.org/10.6084/m9.figshare.21214160 ^41^. This project contains the following underlying data: AcSir2-pinocembrin (PubChem CID: 68071) complex AcSir2-chrysin (PubChem CID: 5281607) complex AcSir2-tectochrysin (PubChem CID: 5281954) complex AcSir2 only Figshare: Raw data for the prediction of the three-dimensional structures. https://doi.org/10.6084/m9.figshare.21214184 ^38^. This project contains the following underlying data: *A. castellanii* Sir2 family protein (AcSir2) AcSir2 protein sequence Result of 3D structure prediction from I-Tasser *A. castellanii* G-protein coupled receptor (AcGPCR) AcGPCR protein sequence Result of 3D structure prediction from I-Tasser *A. castellanii* mannose-binding protein (AcMBP) AcMBP protein sequence Result of 3D structure prediction from I-Tasser Figshare: F1000_raw figures. https://doi.org/10.6084/m9.figshare.21312297 ^39^. This project contains the following underlying data: Figure 3, Figure 4A, Figure 4B, Figure 4C, Figure 5A, Figure 5B, Figure 5C, Figure 6A, Figure 6B, Figure 6C, Figure 7, Figure 8A, Figure 8B, Figure 8C, Figure 8D, Figure 8E, Figure 8F, Figure 8G, Figure 8H, Figure 8I Data are available under the terms of the Creative Commons Attribution 4.0 International license (CC-BY 4.0).
