# Supplementary material for: Targeting Acanthamoeba proteins interaction with flavonoids of Propolis extract by in vitro and in silico studies for promising therapeutic effects
Source: F1000Res. 2023 Mar 27;11:1274. Originally published 2022 Nov 8. [Version 3] doi: 10.12688/f1000research.126227.3 (PMC10015121; doi:10.12688/f1000research.126227.3)
Supplement: Peer review history

# Peer review: Reviewer response for version 2

**DOI:** 10.5256/f1000research.143744.r162657

**Published:** 2023-03-14

**Authors:** Ascel Samba-Louaka

**Affiliations:** 1 Laboratoire Ecologie et Biologie des Interactions, UMR CNRS, Université de Poitiers, Poitiers, France

From my point-of view, I think you can index the paper in the present form.

Is the work clearly and accurately presented and does it cite the current literature?

Yes

If applicable, is the statistical analysis and its interpretation appropriate?

I cannot comment. A qualified statistician is required.

Are all the source data underlying the results available to ensure full reproducibility?

Partly

Is the study design appropriate and is the work technically sound?

Partly

Are the conclusions drawn adequately supported by the results?

Yes

Are sufficient details of methods and analysis provided to allow replication by others?

Yes

Reviewer Expertise:

Cell biology of Acanthamoeba encystment

I confirm that I have read this submission and believe that I have an appropriate level of expertise to confirm that it is of an acceptable scientific standard.

# Peer review: Reviewer response for version 2

**DOI:** 10.5256/f1000research.143744.r162656

**Published:** 2023-02-21

**Authors:** Akm Moyeenul Huq

**Affiliations:** 1 School of Medicine, Department of Pharmacy, University of Asia Pacific, Dhaka, Bangladesh; 2 Bio Aromatic Research Centre, Universiti Malaysia Pahang, Kuantan, Pahang, Malaysia

Recommended for indexing. Satisfactory revisions have been made based on the comments.

Is the work clearly and accurately presented and does it cite the current literature?

Yes

If applicable, is the statistical analysis and its interpretation appropriate?

Yes

Are all the source data underlying the results available to ensure full reproducibility?

Yes

Is the study design appropriate and is the work technically sound?

Yes

Are the conclusions drawn adequately supported by the results?

Yes

Are sufficient details of methods and analysis provided to allow replication by others?

Yes

Reviewer Expertise:

Natural product Pharmacology, Computer aided drug discovery, Herbal medicine and phytherapy

I confirm that I have read this submission and believe that I have an appropriate level of expertise to confirm that it is of an acceptable scientific standard.

# Peer review: Reviewer response for version 1

**DOI:** 10.5256/f1000research.138615.r157400

**Published:** 2023-01-03

**Authors:** Ascel Samba-Louaka

**Affiliations:** 1 Laboratoire Ecologie et Biologie des Interactions, UMR CNRS, Université de Poitiers, Poitiers, France

In this research article, Imran Sama-ae and collaborators highlight the importance of using propolis extracts as inhibitors of Acanthamoeba pathogenicity (encystment, excystment, and adhesion). They have found 3 compounds (pinocembrin, chrysin, and tectochrysin) of propolis extracts that could inhibit *Acanthamoeba* encystment. Using different approaches in structural biology, they suggest that the 3 compounds can bind the *Acanthamoeba castellanii* Sir2 protein (AcSir2) and may be excellent candidates for the development of drugs against *Acanthamoeba*.

 My main comment is about the use of DMSO as a negative control. This control is missing in figures 1 and 2 and table 2. Such control is important as some studies reported DMSO as an inducer of encystment (Siddiqui *et al*., 2016 ^1^ and Tania Martin-Pérez *et al*., 2021 ^2^). We also need this information to understand why the % of encystment is correlated to the concentration of propolis extract (we could expect to have fewer cysts with the highest concentration). The authors have to discuss this point.

**Minor:**

- In which solution the PMSF was solubilized?
- If possible, it could be nice to evaluate the percentage of pinocembrin, chrysin, and tectochrysin in the two other samples of propolis (from Sardasht county and Boroujen city).
- I am not a specialist, so I will not comment on the structural biology aspects. However, can the authors explain why they selected only AcSir2, AcMBP and AcGPCR as host substrates?
- Unless I missed it, the authors should provide the list of the 52 compounds identified in the propolis extract.
- Pinocembrin, chrysin, and tectochrysin seem to bind and inhibit CYP1A2, CYP2C19 and CYP2C9. Isn't this an obstacle to their use as a drug in humans?

Is the work clearly and accurately presented and does it cite the current literature?

Yes

If applicable, is the statistical analysis and its interpretation appropriate?

I cannot comment. A qualified statistician is required.

Are all the source data underlying the results available to ensure full reproducibility?

Partly

Is the study design appropriate and is the work technically sound?

Partly

Are the conclusions drawn adequately supported by the results?

Yes

Are sufficient details of methods and analysis provided to allow replication by others?

Yes

Reviewer Expertise:

Cell biology of Acanthamoeba encystment

I confirm that I have read this submission and believe that I have an appropriate level of expertise to confirm that it is of an acceptable scientific standard, however I have significant reservations, as outlined above.

## References

[1] : The use of dimethyl sulfoxide in contact lens disinfectants is a potential preventative strategy against contracting Acanthamoeba keratitis. *Cont Lens Anterior Eye* .2016;39(5) : 10.1016/j.clae.2016.04.004 389-93 10.1016/j.clae.2016.04.004 27133448

[2] : In Vitro Evaluation of the Combination of Melaleuca alternifolia (Tea Tree) Oil and Dimethyl Sulfoxide (DMSO) against Trophozoites and Cysts of Acanthamoeba Strains. Oxygen Consumption Rate (OCR) Assay as a Method for Drug Screening. *Pathogens* .2021;10(4) : 10.3390/pathogens10040491 10.3390/pathogens10040491 33921633PMC8073477

# Response

**Published:** 2023-01-31

**Authors:** Suthinee Sangkanu

**Affiliations:** Walailak University, Thailand

**Question 1** **:** My main comment is about the use of DMSO as a negative control. (1) This control is missing in Figures 1 and 2 and Table 2. (2) Such control is important as some studies reported DMSO as an inducer of encystment (Siddiqui et al., 2016 and Tania Martin-Pérez et al., 2012). (3) We also need this information to understand why the % of encystment is correlated to the concentration of Propolis extract (we could expect to have fewer cysts with the highest concentration). The authors have to discuss this point.

**Answer** **:** (1) In this study, we diluted the Propolis extract from stock solution (100 mg/mL) that dissolved with 99% DMSO to 1 mg/mL. This dilution made the percentage of DMSO is lower than 1%. So, in untreated condition in all experiments, we used 1% DMSO as a negative control.

 (2) Tania Martin-Pérez et al. (2012) reported the effect of DMSO in *A* *.* *polyphaga* 2961 and *A* *.* *griffini* MYP2004 encystation. 1.25% DMSO induced the encystation but not effect at 0.625%. Siddiqui et al. (2016) showed that 3% DMSO induced amoebae encystation in *A* *.* *castellanii* ATCC 50492. In our negative control (1% DMSO), the results showed that 1% DMSO not effect on encystation in both strains of *A* *.* *castellanii*. (3) In this study Propolis extract at low concentration (1/16 MIC) was able to inhibit the encystation of *A* *.* *castellanii*. The low concentration caused a reduction in the level of encystation of around 80-90%. Example from ATCC30010 showed immature cyst in low concentration (3.9 µg/mL), these immature cysts were destroyed by 0.5% SDS in the counting method. While the high concentrations (31.25 µg/mL) gave a <20% reduction in the encystment levels. And at MIC concentration (62.5 µg/mL), *Acanthamoeba* cells were removed by Propolis extracts. Adaptation to various adverse conditions of *Acanthamoeba* is an essential for the survival under unfavorable conditions. We therefore suggest that low concentration of Propolis extracts is suitable for inhibiting the encystment process.

**Question** **: **In which solution the PMSF was solubilized?

**Answer** **:** PMSF can dissolve in DMSO (Reference: Song F, Han X, Zeng T, Zhang C, Zou C, Xie K. Changes in beclin-1 and micro-calpain expression in tri-ortho-cresyl phosphate-induced delayed neuropathy. Toxicol Lett. 2012;210(3):276-84).

**Question** **:** If possible, it could be nice to evaluate the percentage of pinocembrin, chrysin, and tectochrysin in the two other samples of Propolis (from Sardasht county and Boroujen city).

**Answer:** Thank you for the suggestion. We are sorry to inform you that we are unable to continue this project due mainly to insufficient funds at the present. Nevertheless, we will try to continue another 2 samples of Propolis if we will be able to secure more funds in the near future.

**Question** **: **I am not a specialist, so I will not comment on the structural biology aspects. However, can the authors explain why they selected only AcSir2, AcMBP and AcGPCR as host substrates?

**Answer** **: **AcSir2 was classified as a class-IV sirtuin. This protein exhibited functional SIRT deacetylase activity, localized mainly in the nucleus, and its transcription was upregulated during encystation. *Acanthamoeba* mannose-binding protein (AcMBP) is a virulence factor of the free-living amoeba, which is important for adhesion of the pathogen. G proteins and GPCRs are well known key regulators of cellular communication and cellular functions including cell cycle, mitosis, and proliferation.

**Question** **:** Unless I missed it, the authors should provide the list of the 52 compounds identified in the Propolis extracts.

**Answer** **:** **Supplementary Table 1 ^43^** **. **Chemical compositions of the Propolis extract sampled in Kermanshah city present in Supplementary ^43^.

**Question** **:** Pinocembrin, chrysin, and tectochrysin seem to bind and inhibit CYP1A2, CYP2C19 and CYP2C9. Isn't this an obstacle to their use as a drug in humans?

**Answer** **: **We also added this critical point in the discussion section. See line 807-814

 “However, pinocembrin, chrysin, and tectochrysin seem to bind and inhibit Cytochrome P450, including CYP1A2, CYP2C19 and CYP2C9. Because Cytochrome P450, which is primarily found in the liver, is a crucial detoxification enzyme in the body. This enzyme oxidises xenobiotics to help their excretion. In addition, the cytochrome P450 system can activate and deactivate many drugs ^36^. Therefore, these agents should be used carefully in patients taking other drugs to avoid drug-drug interaction.” A further step that we are contemplating for future work is to work with derivatives of these three compounds and see whether they inhibit the cytochromes or not, while at the same time prove suitable as potential drugs.

# Peer review: Reviewer response for version 1

**DOI:** 10.5256/f1000research.138615.r157123

**Published:** 2023-01-03

**Authors:** Akm Moyeenul Huq

**Affiliations:** 1 School of Medicine, Department of Pharmacy, University of Asia Pacific, Dhaka, Bangladesh; 2 Bio Aromatic Research Centre, Universiti Malaysia Pahang, Kuantan, Pahang, Malaysia

This work focused on the potential of Propolis extract obtained from 3 different places of Iran against *Acanthamoeba*. The activity was screened by MIC calculation and the potentiality of the extract as *Acanthamoeba* inhibitor were accessed in two different phases of life cycle. Phytochemical analysis was done by GC-MS and molecular docking followed by MD simulation were done to see the interaction of phytochemicals with target proteins. The manuscript is well organized and well written. It has the merit to be published in F1000Research journal. However, the following comments should be addressed before indexing:

1. In the MIC determination, chlorohexidine was used as positive control which is a standard drug, but why PMSF was used for anti-ensystation and anti-excystation assay and not chlorohexidine? Does the author want to relate with protease inhibition mechanism of Propolis? If so, then why the author choose different protein targets for docking and MD studies? There is lack of consistency between *in vitro* and *in silico* studies.
2. There is no explanation in the discussion section why Chrysin bound to a different binding site on AcMBP. Which binding site is the active site?
3. There is no standard value for the Pharmacokinetics and toxicity study. Make a footnote under the Table 5.
4. Ligands RMSD and RMSF are not small. The ligand RMSD stays in the trajectory does not necessarily mean the ligand-receptor complex is stable. It can move around the starting pose. The authors should provide a structure overlay comparing the starting pose and a representative snapshot from the MD.

Is the work clearly and accurately presented and does it cite the current literature?

Yes

If applicable, is the statistical analysis and its interpretation appropriate?

Yes

Are all the source data underlying the results available to ensure full reproducibility?

Yes

Is the study design appropriate and is the work technically sound?

Yes

Are the conclusions drawn adequately supported by the results?

Yes

Are sufficient details of methods and analysis provided to allow replication by others?

Yes

Reviewer Expertise:

Natural product Pharmacology, Computer aided drug discovery, Herbal medicine and phytherapy

I confirm that I have read this submission and believe that I have an appropriate level of expertise to confirm that it is of an acceptable scientific standard, however I have significant reservations, as outlined above.

# Response

**Published:** 2023-01-31

**Authors:** Suthinee Sangkanu

**Affiliations:** Walailak University, Thailand

Thank you for all comments. Below are our point by point reply for your further consideration. 

**Question 1** **:** In the MIC determination, chlorohexidine was used as positive control which is a standard drug, but why PMSF was used for anti-encystation and anti-excystation assay and not chlorohexidine?

**Answer** **:** Chlorhexidine is a protein synthesis inhibitor that is positively charged and interacts with the parasite´s negatively charged plasma membrane, resulting in the membrane structure that gives rise to permeability modulation, ionic leakage and cytoplasmic disruptions causing cellular damage and cell death. This drug inhibits cell growth but it has no observable effect on the encystment (Leitsch et al., 2010).

**Question 2** **:** Does the author want to relate with protease inhibition mechanism of Propolis? If so, then why the author chooses different protein targets for docking and MD studies? There is lack of consistency between in vitro and *in silico* studies.

**Answer** **:** From our study Propolis extract is suitable for inhibiting the encystment process like a positive control (PMSF). However, the main mechanism underlying inhibition of encystation by Propolis remain largely unknown. Encystation-related various proteases have been shown to be upregulated and to play an important role in cyst formation. However, how the expression of encystation-related factors is controlled during the encystation process remains unclear. In molecular docking study, sirtuins have been chosen because they are positively associated with a longer life span in some organisms and the potential regulation of pathways mediated by nutrient starvation. They have also been connected to numerous human diseases, thus sirtuin inhibitors have attracted significant attention as potential therapeutics (Joo et al., 2020).

**Question 3** **:** There is no explanation in the discussion section why Chrysin bound to a different binding site on AcMBP. Which binding site is the active site?

**Answer** **:** We added the explanation that why Chrysin is bound to a different binding site on AcMBP in the discussion section following the reviewer's suggestion. See line 782-792

 “After blind docking of the three ligands with the AcMBP, the Chrysin bounded to a different binding site on AcMBP. AcMBP is a virulence factor of the free-living amoeba, which is important for adhesion of the pathogen ^72^. We hypothesize that this protein might have more than one binding site to help them adhere to the surface. To test this hypothesis, we predicted all this protein's binding sites with PrankWeb ^74^. The results are consistent with the hypothesis. The results showed that this protein has more than one pocket and some pockets have similar probability scores (Supplementary Figure 4 and Supplementary Table 1-2 ^43^). It might be possible that this protein has more than one binding site.”

**Question 4** **:** There is no standard value for the Pharmacokinetics and toxicity study. Make a footnote under the Table 5.

**Answer** **:** We added the standard value for the pharmacokinetics and toxicity as a footnote under Table 5 following the reviewer's suggestion. See line 664-649

 “Standard value for the Pharmacokinetics as follow: Caco2 permeability >0.90 = High [High = Good]. Intestinal absorption (human) >30% = Good. Skin Permeability > -2.5 = low [Low = Not Good]. Log VDss < -0.15 = low [Low = Good]. Log VDss > 0.45 = high [High = Not Good]. logBB > 0.3 = readily cross the BBB. logBB < -1 = poorly distributed to the brain. logPS > -2 = penetrate the CNS. logPS < -3 = unable to penetrate the CNS. Minnow toxicity (log LC _50_) < -0.3 = high acute toxicity ^36^.” 

**Question 5** **:** Ligands RMSD and RMSF are not small. The ligand RMSD stays in the trajectory does not necessarily mean the ligand-receptor complex is stable. It can move around the starting pose. The authors should provide a structure overlay comparing the starting pose and a representative snapshot from the MD.

**Answer** **:** We added the picture of the structure compared between the starting pose and every 20 ns (200 frames) snapshot from the MD as Supplementary Figures 1-3 ^43^.
